# The effect of exercise on blood pressure in chronic kidney disease: A systematic review and meta-analysis of randomized controlled trials

**DOI:** 10.1371/journal.pone.0211032

**Published:** 2019-02-06

**Authors:** Stephanie Thompson, Natasha Wiebe, Raj S. Padwal, Gabor Gyenes, Samuel A. E. Headley, Jeyasundar Radhakrishnan, Michelle Graham

**Affiliations:** 1 Department of Medicine, Division of Nephrology, University of Alberta, Edmonton, Alberta, Canada; 2 Department of Medicine, University of Alberta, Edmonton, Alberta, Canada; 3 Department of Medicine, Division of Cardiology Mackenzie Health Science Centre, Edmonton, AB, Canada; 4 Department of Exercise Science and Sport Studies, Springfield College, Springfield, MA, United States of America; 5 Innovation Physical Therapy Edmonton, Alberta, Canada; Universita degli Studi di Perugia, ITALY

## Abstract

**Background and objectives:**

Management of hypertension in chronic kidney disease (CKD) remains a major challenge. We conducted a systematic review to assess whether exercise is an effective strategy for lowering blood pressure in this population.

**Design, setting, participants, and measurements:**

We searched MEDLINE, EMBASE, the Cochrane Library, CINAHL and Web of Science for randomized controlled trials (RCTs) that examined the effect of exercise on blood pressure in adults with non-dialysis CKD, stages 3–5. Outcomes were non-ambulatory systolic blood pressure (primary), other blood pressure parameters, 24-hour ambulatory blood pressure, pulse-wave velocity, and flow-mediated dilatation. Results were summarized using random effects models.

**Results:**

Twelve studies with 505 participants were included. Ten trials (335 participants) reporting non-ambulatory systolic blood pressure were meta-analysed. All included studies were a high risk of bias. Using the last available time point, exercise was not associated with an effect on systolic blood pressure (mean difference, MD -4.33 mmHg, 95% confidence interval, CI -9.04, 0.38). The MD after 12–16 and 24–26 weeks of exercise was significant (-4.93 mmHg, 95% CI -8.83, -1.03 and -10.94 mmHg, 95% CI -15.83, -6.05, respectively) but not at 48–52 weeks (1.07 mmHg, 95% CI -6.62, 8.77). Overall, exercise did not have an effect on 24-hour ambulatory blood pressure (-5.40 mmHg, 95% CI -12.67, 1.87) or after 48–52 weeks (-7.50 mmHg 95% CI -20.21, 5.21) while an effect was seen at 24 weeks (-18.00 mmHg, 95% CI -29.92, -6.08). Exercise did not have a significant effect on measures of arterial stiffness or endothelial function.

**Conclusion:**

Limited evidence from shorter term studies suggests that exercise is a potential strategy to lower blood pressure in CKD. However, to recommend exercise for blood pressure control in this population, high quality, longer term studies specifically designed to evaluate hypertension are needed.

## Introduction

Hypertension is a key determinant of both cardiovascular (CV) events and progressive renal dysfunction.[1–5] For those with moderate to severe chronic kidney disease (CKD), the burden of hypertension is high, with a prevalence of 53% to 95%.[6,7] Although treating hypertension is one of the main priorities in CKD management, control remains suboptimal with less than half of patients attaining recommended blood pressure (BP) targets.[6,8] Medication with or without dietary counseling is the mainstay of blood pressure treatment in CKD; however, antihypertensive drugs are often only partially effective,[9] are costly, frequently confer side effects,[10] and contribute to pill burden.[11] With recent recommendations for tighter control, additional strategies to better manage hypertension in this population are needed.

Exercise is an effective strategy for reducing blood pressure (BP) in non-CKD populations. From systematic reviews of randomized controlled trials (RCTs) in the general population, exercise lowers systolic BP by 3.5 to 6.1 and diastolic BP by 2.5 to 3.0 mmHg.[12–14] The BP reduction with exercise has been greater in people with hypertension with systolic BPs reduced by 8.3 mmHg. The cardiovascular benefits of exercise extend beyond the reduction of traditional cardiovascular risk factors and direct vascular effects, initiated by repeated episodes of exercise-induced shear stress, have been identified.[15] However, many of the mechanisms that mediate these favorable adaptations in vascular structure and function are disrupted in CKD. For instance, CKD is associated with endothelial dysfunction and markedly reduced NO bioavailability as well as increased activity of the sympathetic and renin-angiotensin systems.[16–19]. Therefore, how these disease-specific factors influence the BP response to exercise training is an important question. Several small randomized controlled trials (RCTs) have evaluated the effect of exercise on BP in people with CKD but primarily due to small sample sizes, findings are inconsistent.[20]

Exercise is an appealing strategy for BP control in people with CKD because exercise has shown other benefits in this population[21–23] and also because the attainment of BP targets is potentially cost saving.[24] Therefore, we conducted this systematic review to evaluate the evidence for exercise as a strategy to lower blood pressure in people with non-dialysis dependent CKD (stages 3–5 estimated glomerular filtration rate <60 mL/min/1.73m^2^). As functional and structural changes in the vasculature may precede clinically detectable changes in BP, the effect of exercise on flow mediated dilatation (FMD) and pulse wave velocity was included. We also sought to determine if patient, study level, and exercise factors influence the magnitude of the association.

## Methods and material

This systematic review was conducted and reported according to the Preferred Reporting Items for Systematic Reviews and Meta-Analyses (PRISMA) guidelines.[25]

### Data sources and searches

A comprehensive search designed by a MLIS-trained librarian was performed to identify all randomized controlled trials in adults with non-dialysis CKD comparing an exercise intervention to no exercise intervention or to another form of exercise. We included only trials published in English as full peer-reviewed manuscripts. MEDLINE (1946-present), EMBASE (1974-present), the Cochrane Library, CINAHL (1937-present) and Web of Science (1945-present) were searched for citations up to November 6, 2017. The specific search strategies are provided online as supplementary material (S1 Table). The references of existing systematic reviews were also screened. Two reviewers screened the citations and abstracts. Any trial considered potentially relevant by one or both reviewers was retrieved for further consideration.

### Study selection

Using predetermined eligibility criteria, each potentially relevant trial was independently assessed by two reviewers for inclusion in the review. Disagreements were resolved by consultation with a third party. Randomized trials with adult participants (≥18 years) with stage 3–5 CKD (not on dialysis or with a renal transplant) meeting the following criteria were eligible for inclusion: randomized assignment to one or more exercise interventions, and/or a non-exercise control group; duration of exercise intervention(s) of a minimum of four weeks; and at least one of blood pressure, pulse wave velocity, or flow-mediated dilatation were measured and reported. Trials that only evaluated an acute BP response to exercise were not included. Systolic blood pressure was selected as the primary outcome because it is a stronger predictor of end-stage renal disease (ESRD) than diastolic blood pressure or other measures of arterial stiffness in people with CKD.[1–3,26] Trials with co-interventions were included.

### Data extraction and risk of bias assessment

A single reviewer performed the standardized data extraction method and a second reviewer checked the extracted data for accuracy. The following properties of each trial were recorded: trial characteristics (country, era, design, sample size, follow-up duration, CKD stages and other condition criteria); participants (age, gender, estimated glomerular filtration rate (eGFR), body mass index (BMI), diabetes status, current smoking, and blood pressure medications); exercise prescription (type, intensity, timing and frequency as well as setting and supervision); co-interventions; and outcomes. The outcomes were blood pressure (non-ambulatory and ambulatory systolic, diastolic, mean arterial pressures), pulse wave velocity and flow-mediated dilatation. Authors of included studies were contacted for missing data and for clarification of methods and/or results.

We assessed risk of bias using the Cochrane Collaboration tool[27] and included other items (funding, intention to treat, sample size calculation) also known to be associated with bias.[28–30] Risk of bias was assessed as high, low, or unclear across these items. The overall risk of bias was determined using the following criteria: low risk of bias (low risk of bias for all items; moderate risk (high risk for one item or two or more items unclear); high risk of bias (more than one item as inadequate or two or more items as unclear).[31] Two reviewers assessed the trials independently and resolved any disagreements through discussion.

### Data synthesis and analysis

Data were analyzed using Stata 13.1 (www.stata.com). Missing standard deviations (SD) were imputed using inter-quartile ranges.[32] The difference in means (MD) were used to summarize all (continuous) outcomes. Due to expected diversity between trials, we decided *a priori* to combine results using random effects models. Outcomes were pooled using four categories of follow-up time points: the last available, 12 weeks, 24 weeks, and 48 weeks. Statistical heterogeneity was quantified using the τ^2^ statistic (between-study variance)[33] and the I^2^ statistic. We planned to explore the association between trial and population characteristics (including risk of bias items, all variables in Tables 1–3) and the effect of exercise intervention on the most frequently reported outcome (non-ambulatory systolic blood pressure) where reasonable (at least three trials per category). Univariable weighted (with the inverse of the trial variance) linear meta-regression was used to evaluate for effect modification.[34] Publication bias was assessed by visual inspection of the contour enhanced funnel plot[35] and using weighted regression.[36]

**Table 1:** Population characteristics of included trials.

Trial	Country	Population	Sample size^1^	Mean age, y	Male, %	Mean BMI, kg/m^2^	Mean GFR, mL/min*1.73m^2^	Smoker, %	Diabetes, %	BP,^3^ Sys/Dia	BP meds, %
Ikizler 2018[40]	US	CKD stages 3–4, BMI ≥25	111	57	58	33	42	9	25	132.3/-	-
Aoike 2017[38]	Brazil	CKD stages 3–4, BMI >25	45	56	68	31	27	2	35	130.7/81.9	≥69
Headley 2017[42]	US	GFR 30–59, DM/HTN CKD	49	58	65	36	48	0	46	129.7/79.3	≥63
Kiuchi 2017[44]	Brazil	CKD, HTN	50	58	66	26	43	-	28	125.1/75.4^4^	100
Leehey 2016[41]	US	CKD stages 2–4, type II DM, BMI >30	36	66	100	37	40	17	100	137.1/73.5	100
Greenwood 2015[45]	UK	CKD stages 3–4 (GFR 20–60), progressive decline	20	54	83	28	42	-	≥11	135.0/86.5	≥61
Van Craenenbroeck 2015[46]	Belgium	CKD stages 3–4	48	53	55	28	39	40	10	137.6/81.0	80
Baria 2014[39]	Brazil	CKD stages 3–4, BMI >25	29	52	100	30	28	-	22	MAP 98.2	-
Headley 2014[43]	US	GFR 30–59, DM/HTN CKD	51	58	65	36	48	0	46	129.7/79.3	-
Shi 2014[48]	China	CKD, CVD	21	69	71	-	45	0	33	143.4/88.5	-
Headley 2012[47]	US	CKD stages 2–4 (GFR 15–89)	25	55	-	33	41	-	≥43	121.2/74.5	≥57
Mustata 2011[49]	Canada	CKD stages 3–4 (GFR 15–60)	20	68	65	28^2^	28	-	-	-	-

BMI body mass index, BP blood pressure, CKD chronic kidney disease, CVD cardiovascular disease, GFR glomerular filtration rate, MAP mean arterial blood pressure, UK United Kingdom, US United States

^1^All participants who were randomized

^2^Median

^3^Non-ambulatory blood pressure unless otherwise indicated

^4^24 hour ambulatory blood pressure

A dash indicates that information was not reported.

**Table 2:** Trial and intervention characteristics of included studies.

Trial	Design	Exercise	Intensity	Setting	Supervision	Session length, min	Session frequency, /wk	Follow-up, wk
Ikizler 2018[40]	Factorial (calorie restriction)	Aerobic	60–80% VO_2_ max	Centre	Yes	30–45	3	17
Aoike 2017[38]	Parallel	Aerobic	40–60% VO_2_ max	Home^1^	Mixed	30→50	3	24
				Centre^1^	Yes			
Headley 2017[42]	Parallel	Aerobic	50–60% VO_2_ peak	Centre	Yes	15→55	3	16
Kiuchi 2017[44]	Parallel	Aerobic	2:1 high intensity	Centre	Yes	12→20	5	156
			55–85% HR max	Centre	Yes	30→60	5	
Leehey 2016[41]	Parallel	Aerobic [Resistance]	45–59% VO_2_ peak	Centre then home	Mixed	60 [20–30] then 60 or 30	3 then 3 or 6	52
Greenwood 2015[45]	Parallel	Aerobic [Resistance]	80% HR reserve [80% of 1 rep max]	Mixed	Mixed	2x20→40 [1–2 sets of 10 reps→3 sets of 8–10 reps]	3	52
Van Craenenbroeck 2015[46]	Parallel	Aerobic	90% HR achieved anaerobic threshold	Home	Mixed	10	4/d	13
Baria 2014[39]	Parallel	Aerobic	40–60% VO_2_ max	Home^1^	Mixed	30→50	3	12
				Centre^1^	Yes			
Headley 2014[43]	Matched parallel	Aerobic	50–60% VO_2_ peak	Centre	Yes	15→55	3	16
Shi 2014[48]	Parallel	Tai Chi	NA	Mixed	Mixed	30	3–5	12
Headley 2012[47]	Matched parallel	Aerobic, [Optional resistance]	50–60% VO_2_ peak	Centre	Yes	10→45	3	48
Mustata 2011[49]	Parallel	Aerobic	40–60% VO_2_ peak	Mixed	Mixed	5–20→60	2 plus 3 walking sessions	52

^1^Participants selected their exercise setting, that is, setting was not randomized.

A dash indicates that information was not reported. Arrows indicate duration at the start of the intervention and the targeted progression

**Table 3:** Risk of bias assessment.

Study	Method to randomize described and appropriate (selection bias)	Allocation concealment (selection bias)	Blinding of outcome assessment (detection bias)	Outcome clearly defined (reporting bias)	Sample size calculation	Intention-to-treat	Management of missing data	Incomplete outcome data reported (attrition bias) Withdrawals (%)	Funding
Ikizler 2018[40]	No	Unclear	No	No	Yes^1^	Yes	Exclude	Partial (17)	Government
Aoike 2017[38]	No	Unclear	Unclear	No	Incomplete	No	Exclude	Yes (11)	Foundation, other
Headley 2017[42]	No	Unclear	Unclear	No	No	No	Single imputation	Partial (6)	Government
Kiuchi 2017[44]	No	Unclear	No	Yes	No	No	Exclude	No	Industry
Leehey 2016[41]	Yes	Unclear	Yes	No	Yes	No	Exclude	Yes (11)	Government
Greenwood 2015[45]	Yes	Unclear	Yes	No	Yes^1^	No	Exclude	Yes (20)	Government
Van Craenenbroeck 2015[46]	No	Adequate	Yes	No	Incomplete	No	Exclude	Partial (17)	Foundation, internal
Baria 2014[39]	No	Unclear	Yes	No	Incomplete	No	Exclude	Yes (7)	Foundation
Headley 2014[43]	Yes	Unclear	Unclear	No	Yes	No	Exclude	Partial (10)	Government
Shi 2014[48]	No	Unclear	Unclear	No	No^1^	No	NA	Yes (0)	NR
Headley 2012[47]	No	Unclear	Unclear	No	No^1^	No	Exclude	Yes (16)	Internal
Mustata 2011[49]	Yes	Adequate	Yes	NA	No^1^	Yes	Exclude	Yes (10)	Government

^1^Pilot study

## Results

### Search results

**Fig 1:**
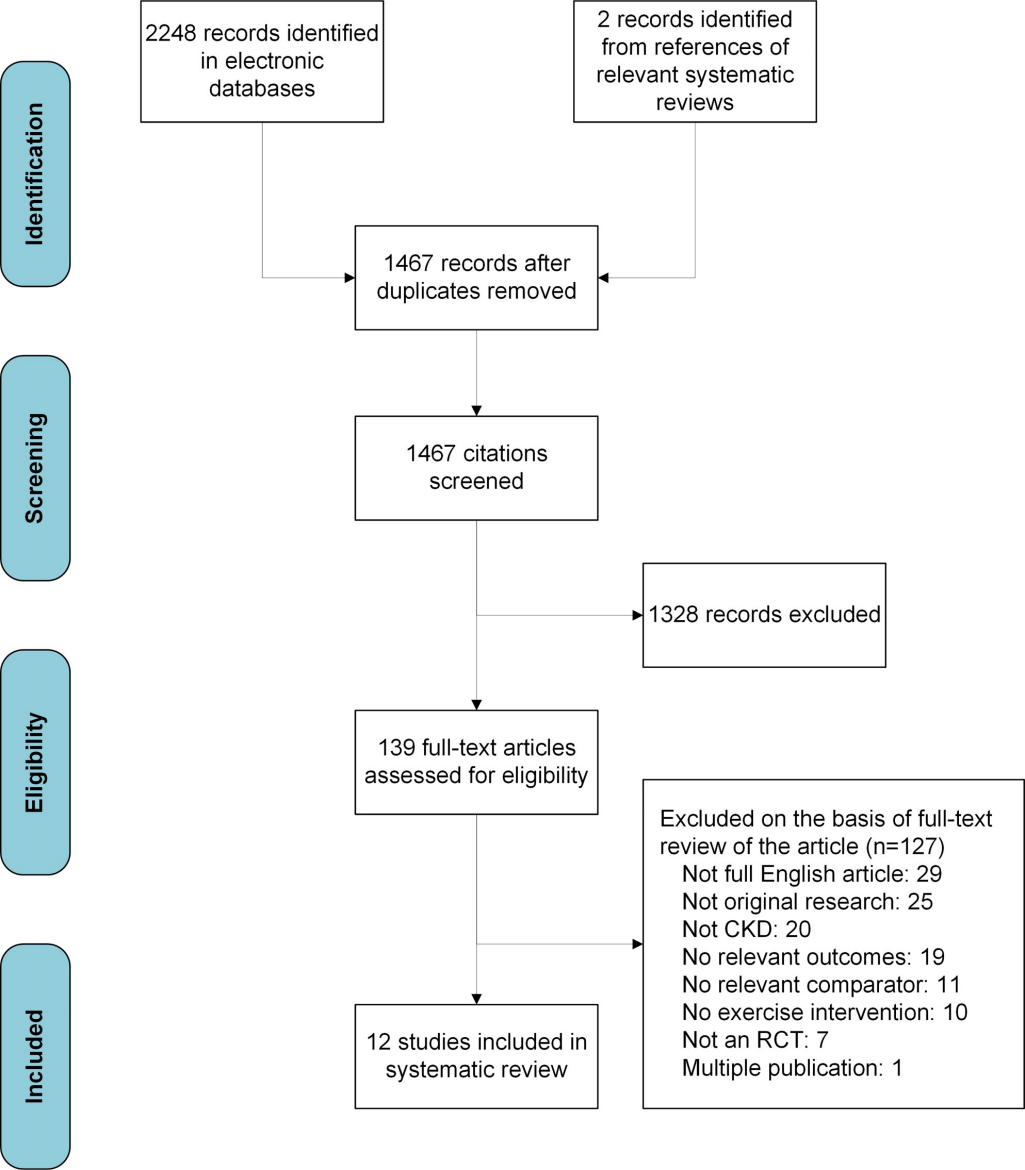
Preferred Reporting Items for Systematic Reviews and Meta-analyses (PRISMA) flow diagram for the systematic review and meta-analysis. CKD chronic kidney disease, RCT randomized controlled trial.

The searches identified 1,467 unique records with two additional records identified from references of existing systematic reviews. After initial screening, 139 articles were retrieved for detailed evaluation (Fig 1) and of these, 127 articles were subsequently excluded, resulting in 12 trials that met the selection criteria. Studies were excluded for the following reasons: 54 were not original research English articles, 20 were not in CKD populations, 19 did not include relevant outcomes, 11 did not have a relative comparator, 10 had no exercise intervention, seven were not randomized trials, and one was a multiple publication of an included trial.[37] Disagreements about the inclusion of trials occurred in 3% of the articles (kappa = 0·80).

### Characteristics of included studies

Six of the 12 trials were conducted in North America (five in the US and one in Canada), three were conducted in Brazil, and one in each Belgium, the UK and China (Table 1). Mean age ranged from 52 to 69 years, and the majority of all participants were consistently male (range 55 to 100%). Mean estimated glomerular filtration rate (eGFR) ranged from 27 to 48 mL/min/1.73m^2^ (median 42 mL/min/1.73m^2^). People with obesity or who were overweight were exclusively recruited in four of the 12 trials (mean BMI for all trials ranged from 26 to 37 kg/m^2^).[38–41] Hypertension[42–44] or diabetes[41] (together with CKD) were the focus of four trials. Baseline mean systolic and diastolic non-ambulatory blood pressures ranged from 121.2 to 143.4 mmHg, and from 74.5 to 88.5 mmHg, respectively. Only seven studies reported the use of BP medications[38,41,42,44–47] and seven trials reported smoking status.[38,40–43,46,48]

Trial and intervention characteristics are shown in Table 2. Eleven of the 12 trials were parallel RCTs. The remaining study was a factorial trial where the second factor was calorie restriction.[40] Study follow-up ranged from 12 to 156 weeks; the median was 21 weeks. All trials included an aerobic intervention except for one where Tai Chi was studied.[48] Resistance exercise was included along with aerobic exercise in two trials[41,45] and optionally in a third trial.[47] Four trials used a high-intensity form of aerobics.[40,44–46] One trial compared a high-intensity interval training to moderate aerobic exercise.[44] Five trials held the exercise sessions entirely in-centre and supervised.[40,42–44,47] The remaining trials had mixed supervision. Of these, one trial had the participants self-select home or centre for exercise;[38] five trials used both centre and home settings;[39,41,45,48,49] and one trial used only home-based exercise sessions.[46] The majority of the trials had participants exercise at least three times per week; the remaining trials had the participants exercise more frequently.

### Risk of bias assessment

All trials were rated with a high risk of bias, mainly due to incomplete reporting (Table 3). Eight trials did not describe randomization methods and only two studies adequately reported allocation concealment. Seven trials did not clearly report blinding of outcome assessment. Only one trial[44] reported blood pressure measurement in accordance with recommended guidelines: non-ambulatory (auscultation or oscillometric) or ambulatory, validation of the instrument, the number of readings, the length of the rest period before the reading(s), and the timing of the reading(s) with respect to the exercise intervention. Four described an *a priori* sample size calculation in complete detail (including two pilot trials). Two reported an intention-to-treat approach to the analysis[40,49] and only one used imputation method for missing data[42] (two trials did not have any missing data). Only one trial did not report information on withdrawals.^22^ Governments, foundations and internal sources funded most trials. Only seven of the trials reported adherence,[40,43,45–47,49] typically as a proportion of the exercise sessions that were completed.

### Effects of exercise on outcomes

#### Effect on non-ambulatory BP

**Fig 2:**
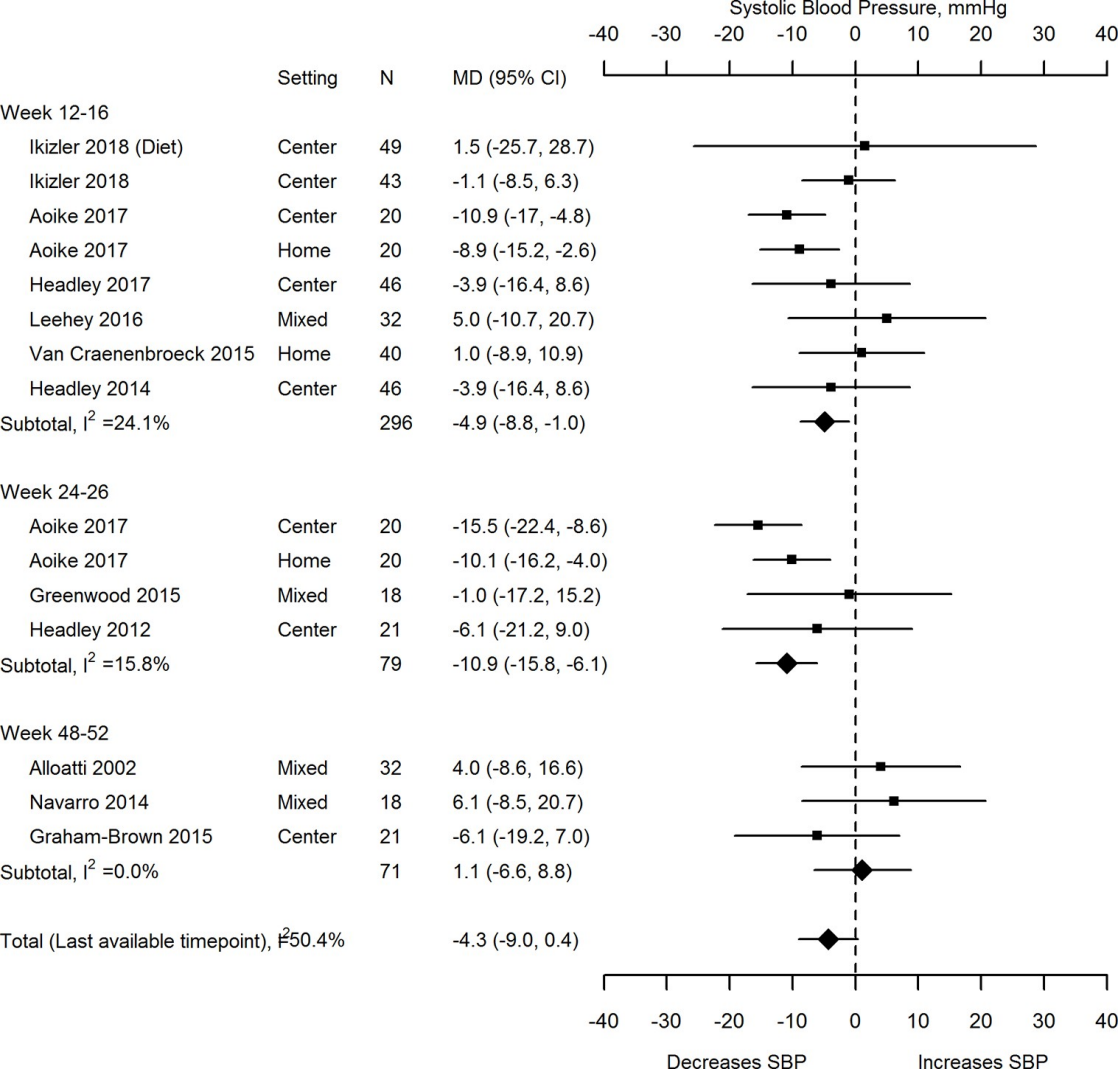
Effects of exercise on non-ambulatory systolic blood pressure: Exercise versus no intervention. CI confidence interval, DBP diastolic blood pressure, MD mean difference, N number of participants, SBP systolic blood pressure.

Ten trials and strata with 335 participants reported non-ambulatory systolic blood pressure (Table 4, Fig 2). Although the direction of the effect favoured a BP-lowering effect of exercise, the mean difference (MD) was not significant (-4.33 mmHg, 95% confidence interval (CI) -9.04, 0.38) using the last available time point; statistical heterogeneity was moderate, I^2^ 50.4%. The MDs favoured exercise at 12–16 and 24–26 weeks (-4.93 mmHg, 95% CI -8.83, -1.03; I^2^ 24.1% in 8 trials and -10.94 mmHg, 95% CI -15.83, -6.05; I^2^ 15.8% in 4 trials, respectively) but not at 48–52 weeks (1.07 mmHg, 95% CI -6.62, 8.77; I^2^ 0.0% in 3 trials). Results for non-ambulatory diastolic blood pressure were not significant except at 24–26 weeks (MD -6.21 mmHg, 95% CI (-10.93, -1.49; I^2^ 37.9%) which favoured exercise (Table 4).

**Table 4:** Meta-analysis: Exercise versus no exercise on blood pressure.

Outcome	Trial or stratum/ Participants	Time point, weeks	MD (95% CI)	I^2^ (%) [T^2^]
*Systolic blood pressure*, *mmHg*				
Non-ambulatory^1^	10/335	Last available	-4.33 (-9.04,0.38)	50.4 [26.32]
Non-ambulatory^1^	8/296	12–16	-4.93 (-8.83,-1.03)	24.1 [7.29]
Non-ambulatory	4/79	24–26	-10.94 (-15.83,-6.05)	15.8 [4.25]
Non-ambulatory	3/71	48–52	1.07 (-6.62,8.77)	0.0 [0.00]
24h ambulatory	2/67	Last available	-5.40 (-12.67,1.87)	0.0 [0.00]
24h ambulatory	1/46	16	-4.38 (-13.25,4.49)	-
24h ambulatory	1/21	24	-18.00 (-29.92,-6.08)	-
24h ambulatory	1/21	48	-7.50 (-20.21,5.21)	-
Day ambulatory	1/46	16	-3.80 (-11.98,4.38)	-
Night ambulatory	1/46	16	-6.30 (-16.35,3.75)	-
*Diastolic blood pressure*, *mmHg*				
Non-ambulatory^1^	8/303	Last available	-1.18 (-4.76,2.40)	60.5 [16.86]
Non-ambulatory^1^	6/264	12–16	-1.46 (-4.60,1.69)	56.5 [9.41]
Non-ambulatory	4/79	24–26	-6.21 (-10.93,-1.49)	37.9 [8.65]
Non-ambulatory	2/39	48–52	2.71 (-4.44,9.84)	0.0 [0.00]
24h ambulatory	2/67	Last available	1.61 (-10.10,13.32)	0.0 [0.00]
24h ambulatory	1/46	16	3.40 (-27.13,33.93)	-
24h ambulatory	1/21	24	-9.00 (-17.71,-0.29)	-
24h ambulatory	1/21	48	1.30 (-11.38,13.98)	-
Day ambulatory	1/46	16	3.30 (-2.78,9.38)	-
Night ambulatory	1/46	16	1.80 (-4.42,8.02)	-
*Mean arterial blood pressure*, *mmHg*				
Non-ambulatory	2/27	12	-12.11 (-15.98,-8.25)	0.0 [0.00]
24h ambulatory	1/46	16	0.30 (-6.29,6.89)	-
Day ambulatory	1/46	16	0.40 (-5.87,6.67)	-
Night ambulatory	1/46	16	-1.20 (-7.97,5.57)	-

24h 24-hour, CI confidence interval, MD mean difference

#### 24-hour ambulatory blood pressure and other BP outcomes

Two trials with 67 participants reported 24-hour ambulatory systolic blood pressure (Table 4).[42,47] The BP-lowering effect of exercise was not significant using the last available follow-up time point (-5.40 mmHg, 95% CI -12.67, 1.87; I^2^ 0.0%). The MD favoured exercise at 24 weeks of follow up -18.00 mmHg, 95% CI (-29.92, -6.08) but was not significant at earlier (16 weeks) and later follow up (48 weeks). Results from one trial with 46 participants[42] reported day and night ambulatory systolic and diastolic blood pressure at 16 weeks. The MDs were not significant.

One trial with 27 participants (and 2 strata–home and centre settings)[39] reported mean arterial pressure at 12 weeks (Table 4). The MD was significant and favoured exercise (-12.1 mmHg, 95% CI -16.0,-8.3; I^2^ 0.0%). Another trial[42] reported 24-hour, day, and night ambulatory mean arterial blood pressures at 16 weeks. The results were not significantly different between exercise and no intervention.

One trial with 50 participants[44] compared a high-intensity exercise to regular aerobic exercise at 52, 104, and 156 weeks both performed 5 times per week (S2 Table). Twenty four-hour ambulatory systolic and diastolic blood pressures were measured. The results generally favoured moderate exercise over the high-intensity exercise (at 156 weeks, systolic BP MD 4.20 mmHg, 95% CI (3.20, 5.20); diastolic BP MD 1.30 mmHg, 95% CI (0.47, 2.13).

#### Pulse wave velocity

Three trials with 104 participants[43,45,46] reported pulse wave velocity (Table 5). The MD was not significant using the last available time points (-0.05 m/s, 95% CI -1.03,0.93; I^2^ 26.5%), nor were any of the results at specific time points. One trial with 20 participants^24^ reported the augmentation index; the MD was also not significant.

**Table 5:** Meta-analysis–Exercise versus no intervention on other vascular outcomes.

Trial or stratum/ Participants	Timepoint, weeks	MD (95% CI)	I^2^ (%) [T^2^]
*Pulse wave velocity*, *m/s*			
3/104	Last available	-0.05 (-1.03,0.93)	26.5 [0.21]
2/86	12–16	0.32 (-0.59,1.24)	0.0 [0.00]
1/18	26	0.50 (-1.26,2.26)	-
1/18	52	-1.20 (-3.06,0.66)	-
*Augmentation index*, *%*			
1/20	52	-0.50 (-7.76,6.76)	-
*Flow-mediated dilation*, *%*			
1/40	12	-0.70 (-2.59,1.19)	-

CI confidence interval, MD mean difference

#### Flow-mediated dilatation

One trial with 40 participants[46] reported flow-mediated dilation at 12 weeks (Table 5). The MD was not significant (-0.70%, 95% CI -2.59,1.19).

#### Metaregression

We regressed non-ambulatory systolic blood pressure on continuous and categorical variables (trial and intervention characteristics, and items of bias), one at a time (Table 6). Two variables significantly modified the effect of exercise on systolic blood pressure (SBP). Trial populations with lower mean baseline eGFRs had larger MDs favouring exercise (difference in MD -0.7 mmHg per 1 mL/min*1.73m^2^ decrease, 95% CI -1.1, -0.2; I^2^ 0.0%). Additionally, trials that did not describe the blood pressure assessment as blinded or measured by a third-party had larger MDs favouring exercise (difference in MD -11.4 mmHg, 95% CI -18.8, -4.0; I^2^ 0.0%). The large MDs from two strata in one trial[38] influenced the pooled results. This trial had a low mean baseline eGFR (27 mL/min/1.73m^2^) and no reported third-party outcome assessment. When we removed this trial from the meta-analysis, the MD shifted to -0.7 mmHg (95% CI -0.2,4.7; I^2^ 0.0%) from -4.33 mmHg.

**Table 6:** Meta-regression of non-ambulatory systolic blood pressure (last available time point).

Covariate	Number of trials or strata	Difference in MD (95% CI)	P	I^2^ (%) [T^2^]
Age (range 53-66y)	10	0.49 per y (-1.28,2.27)	0.54	52.8 [29.43]
Male (range 55–100%)	9	0.15 per % (-0.36,0.65)	0.50	60.7 [36.56]
BMI (range 28–37 kg/m^2^)	10	0.11 per kg/m^2^ (-2.03,2.26)	0.91	54.9 [32.21]
Diabetes (range 10–100%)	8	0.06 per % (-0.23,0.36)	0.62	60.4 [34.16]
Baseline GFR (range 27–48 mL/min*1.73m^2^)	10	0.65 per mL/min*1.73m^2^ (0.20,1.10)	0.01	0.0 [0.00]
Weeks (range 12–52)	10	0.12 per wk (-0.28,0.52)	0.50	54.6 [30.54]
Home/mixed vs center setting	6 vs 4	4.66 (-7.03,16.35)	0.39	53.7 [30.76]
High intensity	4 vs 6	8.94 (-0.74,18.63)	0.07	18.8 [11.24]
Weekly dose (range 70–180 min)	10	-1.88 per 30 min (-7.67,3.91)	0.48	50.0 [28.39]
Supervised vs mixed	6 vs 4	-4.66 (-16.35,7.03)	0.39	53.7 [30.76]
Unclear randomization	7 vs 3	-8.18 (-20.53,4.16)	0.17	41.1 [20.80]
Unclear blinded assessment	5 vs 5	-11.39 (-18.79,-3.98)	0.008	0.0 [0.00]
No/incomplete sample size calculation	5 vs 5	-8.96 (-18.47,0.54)	0.06	17.9 [11.73]
Partial description of LFU	5 vs 5	5.57 (-4.73,15.87)	0.25	36.0 [19.04]
Percentage LFU (range 6.1–20.0%)	10	0.87 per % (-0.47,2.22)	0.17	35.8 [18.94]
Mixed sources of funding vs government	4 vs 6	-8.92 (-17.94,0.10)	0.05	14.5 [9.90]

BMI body mass index, CI confidence interval, GFR glomerular filtration rate, LFU lost to follow-up, MD difference in means, SBP systolic blood pressure

All categorical covariates had ≥3 trials or strata in each category.

#### Publication bias

The funnel plot was mildly asymmetric about the vertical dashed line (the random-effects pooled estimate) likely indicating heterogeneity caused by the two strata from one outlying trial[38] rather than small missing trials with large effect sizes (publication bias) (S1 Fig). Although the weighted regression test did not reach statistical significance (bias 2.2, p = 0.07), the funnel plot is asymmetrical. Without this trial, the funnel plot appeared symmetrical.

## Discussion

Overall, we found that regular exercise was not associated with a significant mean difference in non-ambulatory systolic BP in people with non-dialysis CKD. Exercise was associated with a significant BP-lowering effect at 24 weeks of follow-up, but this difference was not observed at 52 weeks. In the two trials that measured BP using 24-hour ABPM, the overall effect of exercise on systolic BP was also not significant compared to no exercise. Similarly, there was an antihypertensive effect of exercise at 24 weeks that was not detected at 48 weeks. In the interpretation of these findings, it is important to note that the direction and the magnitude of the overall effect of exercise on SBP favoured the intervention. However, our confidence in this finding is limited by the high risk of bias in all of the included trials. Furthermore, the effect moved toward the null when the outlying trial was excluded, and heterogeneity resolved.

Despite the importance of the topic, previous systematic reviews on this topic have primarily included people requiring hemodialysis [50,51] or combined both dialysis and non-dialysis populations in the analysis of blood pressure [52]. From one meta-analysis in people with CKD stages 2–5, exercise training was reported to significantly decrease systolic BP.[53] However, the evidence base for non-dialysis CKD was limited to one small study.[54] In keeping with our findings, a recent metaanalysis of people with stage 3–4 CKD found no difference in BP with aerobic exercise compared to no exercise.[55]

Many of the neurohormonal mechanisms that mediate the beneficial response to exercise are altered in CKD. For example, several studies have shown that the physiologic elevation in heart rate and BP that normally occur during exercise is exaggerated in people with CKD, potentially due to the lower bioavailability of nitrous oxide and the sympathetic nervous system over-activation in this population.[56–58] Whether these alterations in endothelial function and neurohormonal systems influence the longer-term training response to exercise in CKD is not known. However, in other conditions associated with endothelial dysfunction, such as cardiovascular disease and diabetes, exercise training has been associated with improvements in endothelial function.[59,60] Although we did not detect differences in flow-mediated dilatation in this review, the data were limited to one study that used a moderate intensity intervention over 12 weeks. Other reviews on exercise and endothelial function have reported differential responses to exercise and it is possible that the exercise prescription and disease-related factors influence vascular adaptations. In one review of both healthy and clinical populations, greater improvements in FMD were shown with high intensity exercise whereas in type 2 diabetes, low to moderate intensity exercise showed greater effects on improving FMD than moderate to high intensity exercise.[59,61] In healthy populations, vascular function adaptations to exercise training have been observed within eight weeks;[62] however, it is plausible that longer or more frequent interventions may be needed to reverse advanced endothelial dysfunction.

Our finding that the magnitude of the BP reduction appeared greater with more advanced kidney dysfunction is interesting and warrants further investigation. It is known that PWV is negatively associated with renal function [63] and the findings from several studies in select populations suggest that advanced vascular stiffness modifies the vascular adaptations to exercise training. For example, the BP response to exercise has been more modest in older adults with hypertension and also did not change measures of aortic stiffness.[64–66] This effect is highly relevant to the CKD population as CKD disproportionately affects older adults.[7] Furthermore, CKD is characterized by pronounced and accelerated vascular calcification, independent of age.[67] In one observational study in people with CKD, higher physical activity levels were associated with a lower risk of CV events in those age 65 years and younger, but not older.[68] Therefore, whether aortic stiffness modifies the BP response to exercise training in people with CKD is an important questions for future research.

Antihypertensive use at baseline in the majority of included studies were incomplete but estimated as high overall, ranging from 57–100%. That antihypertensive medication use attenuates the BP response to exercise was suggested by one author as a potential explanation for the null findings.[42] Although, data from RCTs to refute this hypothesis are limited, in one study of people with resistant hypertension (prescribed a mean of 4 antihypertensives at baseline) and without CKD, 24-hour systolic ABPM was reduced by 5.4 (±12.2) mmHg after 12 weeks of aerobic exercise.[69] The question of whether resistance to antihypertensives predicts resistance to exercise is highly germane to the CKD population. In contrast to essential hypertension, the progression of CKD is associated with worsening BP control, primarily due to fluid overload and multiple medications including a diuretic are frequently prescribed.[70] Although the mechanism has not been fully elucidated, it is plausible that high extracellular fluid volume (and thus, cardiac output) due to more severe renal dysfunction may offset any beneficial adaptations in total peripheral resistance. As inter-individual variation in the chronic BP response to exercise training is phenomenon that is recognized in other populations,[71] factors that may modify the BP response to exercise, such as fluid balance should be explored in CKD.

Our findings highlight several important considerations for future studies in this area. Only one trial adequately reported blood pressure and the methods for measurement between studies were highly variable. Variable measurement is known to result in clinically significant differences in BP readings. To improve the accuracy and comparability of findings, standardization of blood pressure measurement is necessary.[72,73] We recommend that in future studies, investigators report blood pressure according to recommended standards for research.[74] Compared to people with normal renal function, 24-hour variation in SBP is higher in people with CKD.[75,76] This variability along with the known limitations of office blood pressures (e.g. white coat effect and measurement error from improper technique) underscores the importance of using 24-hour ambulatory monitoring in efficacy trials using BP as a main outcome variable.[77] In addition, adherence to exercise was only reported in seven trials. Although adherence appeared adequate at greater than 70%, the most common method of reporting was the number of exercise sessions completed or attended. Only one study reported the exercise that was actually performed by participants.[46] To interpret findings that could be attributed to non-adherence rather than lack of efficacy, (i.e. our finding of a BP-lowering effect at 24–26 weeks but not at longer follow up), information on adherence including attained intensity and duration of exercise, completed sessions, and changes in fitness are necessary. Finally, future studies should include information on important confounders, including alteration of dietary sodium intake and adjustment of antihypertensive medications. It is important to consider that as no study reported antihypertensive dose adjustments as an outcome, it is possible that differential adjustment of BP medications could explain the null findings. Given the high cost and use of antihypertensives among this population, changes in the dose or number of anithypertensives are a highly relevant outcome.

To our knowledge, this is the largest meta-analysis of exercise on blood pressure in people with non-dialysis dependent CKD. In order to provide accurate estimates of exercise training, we excluded studies that did not apply a co-intervention to a comparator group and reported our findings according to the method of BP ascertainment. However, there are limitations to this study. First, only three trials in this review were specifically designed to evaluate the effect of exercise on BP. The baseline BP in the study populations was also well controlled, which may have lessened the response to the intervention. Second, heterogeneity had a moderate impact on the overall analysis; however, when the outlier study was removed, this resolved. Heterogeneity was also low when the BP response was analysed by time point. Third, in contrast to data from the non-CKD population,[78,79] we found that higher intensity exercise was not associated with a greater anti-hypertensive effect; however, most studies evaluated a moderate intensity aerobic intervention and we were unable to fully explore the BP response to different exercise prescriptions. Lastly, we only included published studies and it is possible that our analysis was underpowered to detect publication bias.

In conclusion, the evidence base to show that exercise is an effective strategy for reducing BP in people with CKD is limited by small studies with a high risk of bias. Although current guidelines encourage exercise counselling for cardiovascular health in people with CKD, more evidence that exercise is an effective strategy for lowering BP is needed to inform guidelines that clearly prioritize exercise in the delivery of CKD care. For renal health care providers, a high degree of confidence in the efficacy of exercise will be needed to expand the current paradigm of BP management to include exercise counselling and to justify allocating the additional resources that will be required to increase physical activity in this highly sedentary population.

## Supporting information

S1 Fig Funnel plot of non-ambulatory systolic blood pressure: exercise versus no intervention. Each trial’s precision (the inverse of the standard error of each trial’s effect estimate) is plotted against each trials’ effect estimate (mean difference).
(TIF)

S1 Table Search strategies. (PDF)

S2 Table Meta-analysis–High intensity exercise versus regular intensity exercise. CI confidence interval, MD mean difference.
(PDF)

S1 File Thompson-SR Ex and BP on CKD-PRISMA 2009 checklist. (DOCX)
