# Correction: The effect of exercise on blood pressure in chronic kidney disease: A systematic review and meta-analysis of randomized controlled trials

**DOI:** 10.1371/journal.pone.0233869

**Published:** 2020-05-21

**Authors:** Stephanie Thompson, Natasha Wiebe, Raj S. Padwal, Gabor Gyenes, Samuel A. E. Headley, Jeyasundar Radhakrishnan, Michelle Graham

There are errors in Fig 2. The studies listed in Week 48–52 are incorrect. The authors have provided a corrected version here.

**Fig 2:**
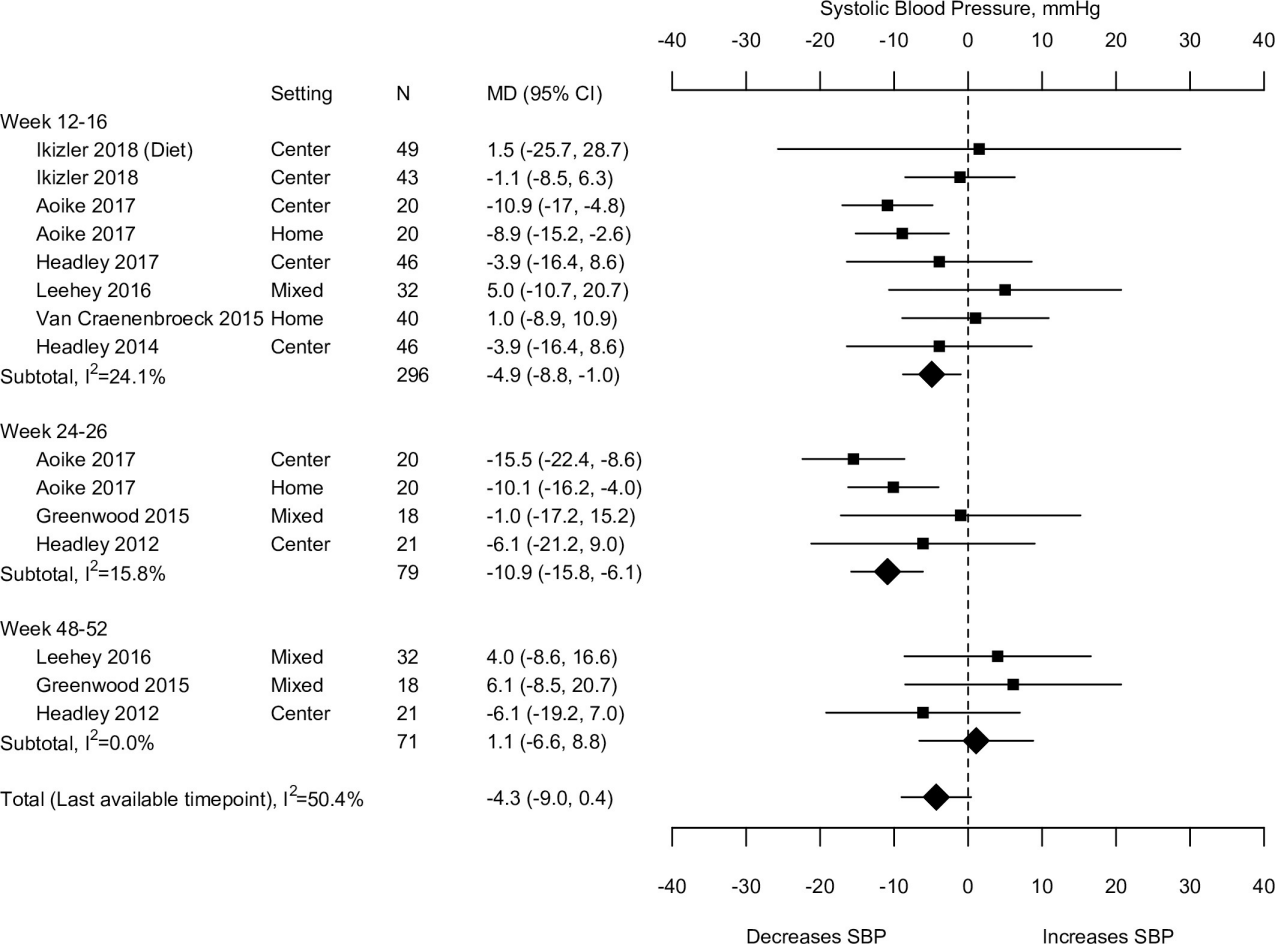
Effects of exercise on non-ambulatory systolic blood pressure: Exercise versus no intervention. CI confidence interval, DBP diastolic blood pressure, MD mean difference, N number of participants, SBP systolic blood pressure.
