# Supplementary figures and images for: The effect of exercise on blood pressure in chronic kidney disease: A systematic review and meta-analysis of randomized controlled trials
Source: PLoS One. 2019 Feb 6;14(2):e0211032. doi: 10.1371/journal.pone.0211032 (PMC6364898; doi:10.1371/journal.pone.0211032)

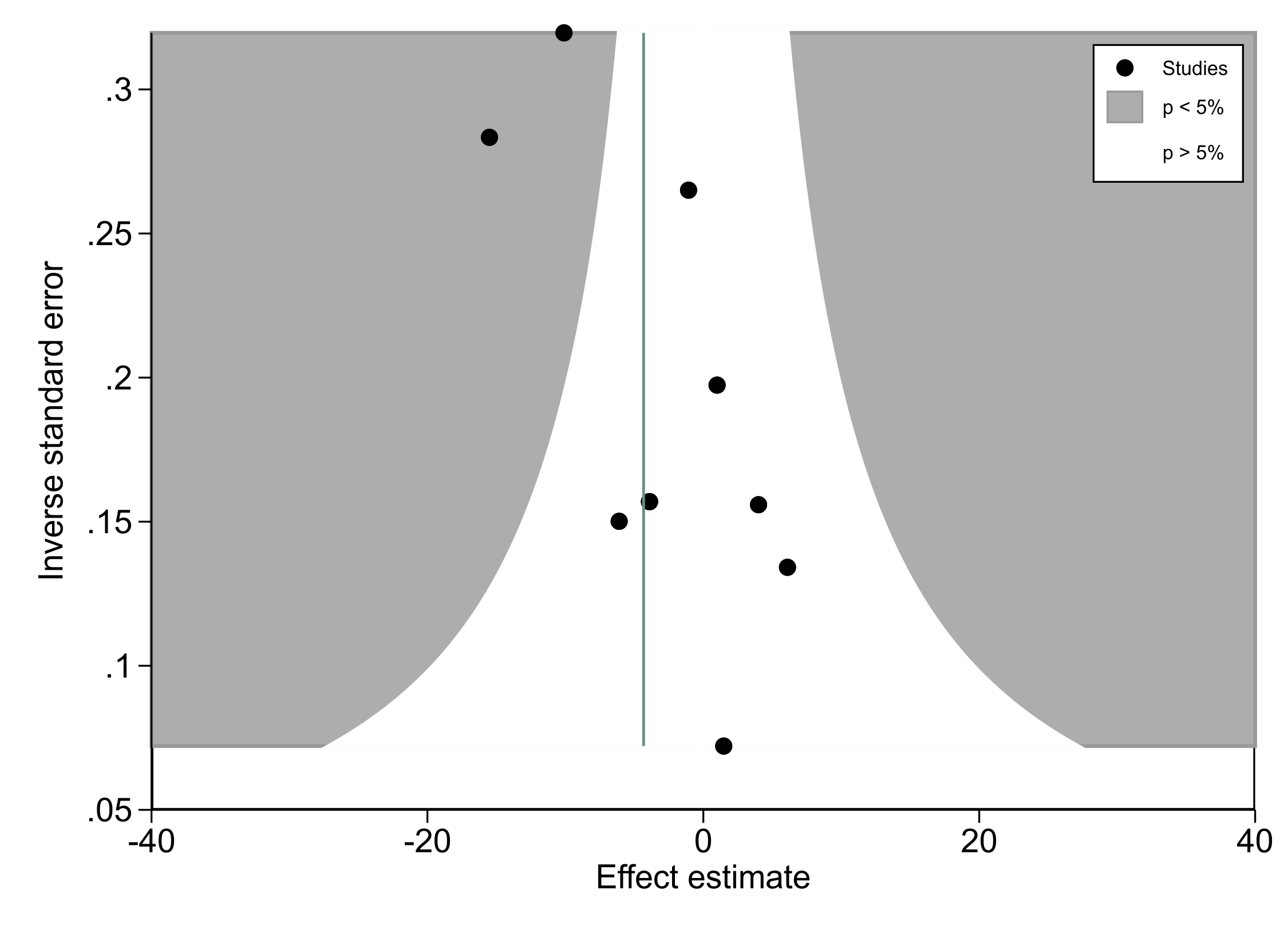

Supplement: S1 Fig — Each trial’s precision (the inverse of the standard error of each trial’s effect estimate) is plotted against each trials’ effect estimate (mean difference). (TIF) [file pone.0211032.s001.tif]
