# Supplementary material for: The effect of exercise on blood pressure in chronic kidney disease: A systematic review and meta-analysis of randomized controlled trials
Source: PLoS One. 2019 Feb 6;14(2):e0211032. doi: 10.1371/journal.pone.0211032 (PMC6364898; doi:10.1371/journal.pone.0211032)
Supplement: S1 Table — (PDF) [file pone.0211032.s002.pdf]

S1 Table. Search strategies

Database and platform	Search Strategies	Date and coverage
MEDLINE (OVID)	1. exp Exercise Therapy/<br>2. exp Exercise/ or exp Sports/<br>3. physical fitness/ or cardiorespiratory fitness/<br>4. exercise*.ti,kf.<br>5. exercise*.ab. /freq=2<br>6. ((resistance or weight) adj (training or program*)).tw,kf.<br>7. (physical adj (fitness or rehabilitation or activit*)).tw,kf.<br>8. (walking or yoga or swimming or cycling or bicycling or weight lifting or jogging).tw,kf.<br>9. or/1-8<br>10. renal insufficiency/ or exp renal insufficiency, chronic/<br>11. (chronic kidney disease or chronic renal disease).tw,kf.<br>12. (end stage adj (renal failure or kidney failure or renal disease or kidney disease)).tw,kw.<br>13. (CKF or CKD or ESRF or ESKF or ESKD or ESRD or ((CRD or CRF) and (kidney or renal or nephrology))).tw,kf.<br>14. or/10-13<br>15. 9 and 14<br>16. randomized controlled trial.pt.<br>17. clinical trial.pt.<br>18. randomi?ed.ti,ab.<br>19. placebo.ti,ab.<br>20. dt.fs.<br>21. randomly.ti,ab.<br>22. trial.ti,ab.<br>23. groups.ti,ab.<br>24. or/16-23<br>25. animals/<br>26. humans/<br>27. 25 not (25 and 26)<br>28. 24 not 27<br>29. 15 and 28<br>30. (editorial or guideline or letter or news or newspaper article or practice guideline).pt.<br>31. 29 not 30<br>32. exp Child/<br>33. exp adults/<br>34. 32 not (32 and 33)<br>35. 31 not 34<br>36. remove duplicates from 35	Ovid MEDLINE® Epub ahead of print, In-process & other non-indexed citations, Ovid MEDLINE® Daily, and Ovid MEDLINE® 1946 to Present<br><br>November 6, 2017

Database and platform	Search Strategies	Date and coverage
	37. limit 36 to english language	
EMBASE (OVID)	1. exp kinesiotherapy/<br>2. exp *Exercise/ or exp sport/<br>3. fitness/ or exp physical activity/<br>4. exercise*.ti,kw.<br>5. exercise*.ab. /freq=2<br>6. ((resistance or weight) adj (training or program*)).tw,kw.<br>7. (physical adj (fitness or rehabilitation or activit*)).tw,kw.<br>8. (walking or yoga or swimming or cycling or bicycling or weight lifting or jogging).tw,kw.<br>9. or/1-8<br>10. chronic kidney failure/ or "chronic kidney disease-mineral and bone disorder"/<br>11. (chronic kidney disease or chronic renal disease).tw,kw.<br>12. (end stage adj (renal failure or kidney failure or renal disease or kidney disease)).tw,kw.<br>13. (CKF or CKD or ESRF or ESKF or ESKD or ESRD or ((CRD or CRF) and (kidney or renal or nephrology))).tw,kw. [Both CRF and CRD were bringing in many irrelevant citations on various things including chronic respiratory failure and chronic respiratory disease]<br>14. or/10-13<br>15. 9 and 14<br>16. exp clinical trial/<br>17. randomi?ed.ti,ab.<br>18. placebo.ti,ab.<br>19. dt.fs.<br>20. randomly.ti,ab.<br>21. trial.ti,ab.<br>22. groups.ti,ab.<br>23. or/16-22<br>24. (exp vertebrate/ or animal/ or exp experimental animal/ or nonhuman/ or animal.hw.) not exp human/<br>25. 23 not 24<br>26. 15 and 25<br>27. (conference or conference abstract or conference paper or editorial or erratum or letter).pt.<br>28. 26 not 27<br>29. exp child/<br>30. exp adult/<br>31. 29 not (29 and 30)	EMBASE 1974 to 2017 November 5<br><br>November 6, 2017

Database and platform	Search Strategies	Date and coverage
	32. 28 not 31<br>33. limit 32 to english language<br>34. remove duplicates from 33	
Cochrane Library	1. [mh "Exercise Therapy"]<br>2. [mh Exercise]<br>3. [mh ^"physical fitness"] or [mh ^"cardiorespiratory fitness"]<br>4. (exercise*):ti,ab,kw<br>5. ("resistance training"):ti,ab,kw or ("weight training"):ti,ab,kw or ("resistance program*"):ti,ab,kw or ("weight program*"):ti,ab,kw<br>6. ("physical fitness" or "physical rehabilitation" or "physical activit*"):ti,ab,kw<br>7. (walking or yoga or swimming or cycling or bicycling or "weight lifting" or jogging):ti,ab,kw<br>8. #1 or #2 or #3 or #4 or #5 or #6 or #7<br>9. [mh ^"renal insufficiency"] or [mh "renal insufficiency, chronic"]<br>10. ("chronic kidney disease" or "chronic renal disease"):ti,ab,kw<br>11. ("end stage kidney disease" or "end stage renal disease" or "end stage kidney failure" or "end stage kidney failure"):ti,ab,kw<br>12. (CKF or CKD or ESRF or ESKF or ESKD or ESRD):ti,ab,kw<br>13. #9 or #10 or #11 or #12<br>14. #8 and #13	November 6, 2017
CINAHL (EBSCOhost)	S1 (MH "Renal Insufficiency, Chronic+") OR (MH "Renal Insufficiency")<br>S2 "chronic kidney disease" or "chronic renal disease"<br>S3 "end stage renal" OR "end stage kidney"<br>S4 CKF or CKD or ESRF or ESKF or ESKD or ESRD OR CRD or CRF<br>S5 S1 OR S2 OR S3 OR S4<br>S6 (MH "Therapeutic Exercise+") OR (MH "Exercise+") OR (MH "Physical Fitness+") OR (MH "Cardiorespiratory Fitness") OR (MH "Sports+") OR (MH "Physical Activity")<br>S7 exercise OR (physical N0 (fitness OR rehabilitation OR program*)) OR ((resistance or weight) N0 (training or program*)) OR (walking or yoga or swimming or cycling or bicycling or "weight lifting" or jogging)<br>S8 S6 OR S7<br>S9 S5 AND S8<br>S10 (MH "Clinical Trials+")<br>S11 PT Clinical trial<br>S12 clinic* n1 trial*	November 6, 2017

Database and platform	Search Strategies	Date and coverage
	S13 (singl* n1 blind*) OR (singl* n1 mask*)<br>S14 doubl* n1 blind* OR doubl* n1 mask*<br>S15 tripl* n1 blind* OR tripl* n1 mask*<br>S16 trebl* n1 blind* OR trebl* n1 mask*<br>S17 (trebl* n1 blind*) OR (trebl* n1 mask*)<br>S18 randomi* control* trial*<br>S19 (MH "Random Assignment")<br>S20 random* allocat*<br>S21 placebo*<br>S22 (MH "Placebos")<br>S23 allocat* random*<br>S24 S10 OR S11 OR S12 OR S13 OR S14 OR S15 OR S16 OR S17 OR S18 OR S19 OR S20 OR S21 OR S22 OR S23<br>S25 S9 AND S24<br>S26 S9 AND S24 Limiters - English Language	
Web of Science	#1. TS=("exercise*" OR "physical fitness" OR "weight training" OR "weight program*" OR "resistance training" OR "resistance program" OR "physical rehabilitation" OR "physical activit*") OR TS=(walking or yoga or swimming or cycling or bicycling or "weight lifting" or jogging) Indexes=SCI-EXPANDED, SSCI, CPCI-S, CPCI-SSH, BKCI-S, BKCI-SSH, ESCI Timespan=All years<br>#2. TS=("chronic kidney disease" OR "chronic renal disease" OR "end stage kidney" OR "end stage renal") OR TS=(CKF OR CKD OR ESRF OR ESKF OR ESKD OR ESRD) OR TS=(CRD AND (kidney OR renal OR nephrology)) OR TS=(CRF AND (kidney OR renal OR nephrology)) Indexes=SCI-EXPANDED, SSCI, CPCI-S, CPCI-SSH, BKCI-S, BKCI-SSH, ESCI Timespan=All years<br>#3. #2 AND #1<br>Indexes=SCI-EXPANDED, SSCI, CPCI-S, CPCI-SSH, BKCI-S, BKCI-SSH, ESCI Timespan=All years<br>#4. TS=("clinical trial*" OR "controlled trial*" OR "single blind*" OR "double blind*" OR "triple blind*" OR RCT) Indexes=SCI-EXPANDED, SSCI, CPCI-S, CPCI-SSH, BKCI-S, BKCI-SSH, ESCI Timespan=All years<br>#5. #4 AND #3<br>Indexes=SCI-EXPANDED, SSCI, CPCI-S, CPCI-SSH, BKCI-S, BKCI-SSH, ESCI Timespan=All years	Web of Science Advanced Search<br><br>November 6, 2017
