# Supplementary material for: The effect of exercise on blood pressure in chronic kidney disease: A systematic review and meta-analysis of randomized controlled trials
Source: PLoS One. 2019 Feb 6;14(2):e0211032. doi: 10.1371/journal.pone.0211032 (PMC6364898; doi:10.1371/journal.pone.0211032)
Supplement: S2 Table — CI confidence interval, MD mean difference. (PDF) [file pone.0211032.s003.pdf]

S2 Table. Meta-analysis – High intensity exercise versus regular intensity exercise

Outcome	Trial/ Participants	Timepoint, weeks	MD (95% CI)	I^2 (%) [T^2]
Systolic blood pressure, mmHg				
24h ambulatory	1/50	52	3.50 (2.34,4.66)	-
24h ambulatory	1/50	104	3.50 (2.34,4.67)	-
24h ambulatory	1/50	156	4.20 (3.20,5.20)	-
Diastolic blood pressure, mmHg				
24h ambulatory	1/50	52	0.90 (-0.08,1.88)	-
24h ambulatory	1/50	104	0.90 (-0.14,1.94)	-
24h ambulatory	1/50	156	1.30 (0.47,2.13)	-

CI confidence interval, MD mean difference
